# Supplementary material for: Surface Microbiome Profiling of Dental Elevators Using Third‐Generation Sequencing: Implications for Infection Control in Dental Practice
Source: Microbiologyopen. 2025 Dec 8;14(6):e70178. doi: 10.1002/mbo3.70178 (PMC12685768; doi:10.1002/mbo3.70178)
Supplement: Supplementary file 1 — customer_backup. [file MBO3-14-e70178-s001.zip › customer_backup/customer_backup/taxa_summary/krona/samples/M15.Krona.html]

Javascript must be enabled to view this page.

members
magnitude
magnitudeUnassigned

M15.krona

55140

55013

571

526

522

214

214

214

55

55

55

253

253

253

4

4

4

4

45

45

45

45

1

44

2913

86

86

86

86

86

1252

1252

1128

663

99

562

2

465

256

209

124

2

1

1

3

3

2

2

30

29

1

74

74

12

12

1

1

965

783

781

57

57

150

150

36

36

2

2

471

471

65

65

2

2

2

3

1

1

1

1

1

1

1

1

1

179

24

8

8

16

16

47

47

3

1

43

73

4

1

3

19

14

5

50

2

41

7

35

1

1

5

5

29

16

13

80

56

1

1

1

20

13

3

2

3

5

4

4

2

2

1

1

35

1

1

27

27

7

7

24

12

12

2

8

2

12

1

1

11

11

526

81

81

2

2

79

1

3

12

1

20

42

3

3

3

3

49

39

7

1

6

31

18

3

3

1

2

1

3

1

1

10

7

7

1

1

2

2

2

2

1

1

1

1

1

1

1

1

3

3

1

1

2

2

6

2

2

2

2

2

1

1

2

2

2

15

5

2

2

3

3

10

3

3

2

2

5

5

188

188

188

1

80

14

3

90

177

9

1

1

8

8

63

3

3

24

24

13

13

2

1

1

21

4

3

2

12

14

12

12

2

2

91

4

4

3

3

1

1

1

1

3

1

2

79

79

1

1

1

1

4

4

4

4

1

2

1

159

159

159

159

6

6

8

8

24

24

12

12

109

109

78

70

70

70

4

4

25

4

21

27

27

14

14

8

8

8

8

8

992

992

992

992

992

992

31

31

31

27

8

8

16

16

3

1

2

4

4

2

2

278

278

278

165

165

1

12

152

4

4

4

109

109

109

3

3

3

3

3

3

229

219

7

7

4

4

1

1

2

2

212

11

11

11

3

1

1

2

2

2

2

2

180

180

180

10

4

4

1

1

5

5

6

6

6

10

10

1

1

1

1

1

1

2

2

2

2

2

2

4

4

4

1

1

1

1

1

1

4402

26

26

26

15

15

9

1

8

2

2

1558

72

72

72

72

1417

89

89

89

30

3

3

11

11

16

16

1298

53

46

7

40

40

201

181

20

605

77

120

140

268

151

151

248

87

161

69

69

69

57

12

465

465

465

417

417

48

48

1825

1825

563

4

4

53

53

1

1

3

3

82

82

17

17

403

393

10

40

26

26

14

14

170

170

170

1052

1052

17

881

34

120

527

521

486

59

59

427

427

4

4

4

19

19

19

12

12

12

3

3

3

3

3

3

3

3

1

1

1

1

1

11

11

11

11

11

11

21

21

21

8

8

8

13

13

13

146

4

4

4

4

4

9

9

9

9

9

125

125

125

1

1

124

124

8

8

8

8

8

1198

59

56

56

56

56

3

3

1

1

2

2

2

1

1

1

1

1

1

1

1

1064

1064

1064

391

391

673

1

255

414

3

61

61

61

61

61

12

5

1

1

1

3

3

3

1

1

1

7

7

7

7

33

33

33

6

6

6

6

6

6

11

11

11

10

10

10

3

3

3

3

3

3

474

5

5

5

1

1

2

2

1

1

1

1

26

26

26

26

26

443

443

443

1

1

439

47

389

3

1

1

2

2

540

85

85

11

11

11

74

74

74

136

127

83

83

83

5

5

5

39

39

39

9

9

9

4

5

317

150

149

3

1

2

146

1

145

1

1

1

1

1

1

1

161

101

5

5

85

85

11

11

44

20

1

19

24

24

16

16

4

12

5

5

5

5

2

2

2

2

2

24

24

24

21

21

21

3

3

1

2

37

27

1

1

1

1

26

25

25

25

1

1

1

7

7

7

7

7

3

3

3

3

3

17878

1278

1

1

1

1

15

15

15

14

1

10

10

9

3

6

1

1

1

1

1

1

11

11

11

11

13

13

13

13

69

69

9

5

4

3

3

3

3

2

1

1

1

1

22

13

4

5

25

1

5

9

2

1

1

1

3

1

1

4

2

2

16

16

16

16

517

156

94

94

62

40

22

2

2

2

4

4

4

2

2

2

83

71

57

14

9

9

3

3

1

1

1

90

78

1

77

12

12

10

1

1

3

3

6

6

107

10

10

1

1

9

8

1

8

3

5

4

4

30

21

9

42

42

3

1

2

3

1

1

1

1

1

1

1

1

1

58

26

26

1

1

29

2

18

9

2

2

85

1

1

1

84

1

1

82

81

1

1

1

1

1

1

1

168

168

11

4

6

1

154

41

30

83

3

3

1

1

1

1

1

1

1

1

17

6

6

6

11

11

11

24

24

1

1

6

6

2

1

1

12

1

9

1

1

3

1

2

87

1

1

1

75

75

75

11

11

11

241

241

1

1

12

12

12

12

2

2

3

2

1

4

4

33

3

8

22

8

8

19

8

11

19

3

6

10

106

106

14

14

3

3

5

5

16600

11

11

11

8

3

6

6

6

6

2

2

2

2

1570

1

1

1

995

988

876

112

7

7

224

196

196

22

22

6

6

2

2

2

2

2

2

344

205

205

138

138

1

1

2

2

2

9063

8

8

8

113

113

113

23

2

2

1

1

13

13

7

7

4

4

4

7

6

5

1

1

1

5

5

5

1

1

1

10

2

2

8

8

1

1

1

3937

2

2

1

1

1

1

275

275

11

7

4

1386

1

1385

1

1

4

4

2256

2256

4954

1

1

4845

4845

94

8

24

44

18

14

2

3

6

3

2540

2

1

1

1

1

2538

2494

836

1658

7

7

37

37

3

3

3

3

3

3

3

3

61

30

30

30

31

31

31

1082

635

635

635

447

447

447

24

24

24

24

20

11

11

11

9

9

9

80

1

1

1

79

74

74

1

1

4

1

2

1

26

26

26

26

10

4

4

4

6

6

6

156

143

143

86

41

16

13

13

13

11

11

8

8

3

3

457

20

20

20

9

9

9

9

9

7

2

396

38

27

1

10

57

30

27

271

136

81

54

30

30

1

1

1

7

3

3

4

1

3

2

2

2

13

7

7

6

6

49

39

39

39

10

10

10

11

11

11

11

648

648

2

2

646

144

58

444

17

17

17

17

28

17

4

4

13

13

11

11

11

48

48

48

48

6

6

6

6

198

15

15

15

32

32

32

151

151

151

213

109

109

109

104

5

5

36

36

63

10

53

91

91

59

7

52

32

32

14

14

14

8

6

152

152

152

152

51

51

51

51

51

51

640

405

405

405

2

2

400

1

399

3

3

235

18

12

12

12

6

6

6

7

5

5

5

2

2

2

1

1

1

1

9

9

9

9

186

8

8

8

17

17

17

161

1

1

160

158

2

3

3

3

3

11

11

11

1

10

6

6

6

6

6

3

3

107

24

19

19

19

19

5

5

5

5

1

1

1

1

1

77

17

17

17

17

14

14

14

2

12

4

4

4

4

42

42

27

27

15

15

5

5

5

5

5

18160

5888

3

3

2

2

1

1

19

19

17

17

2

2

16

16

16

16

11

11

11

11

2552

2551

119

18

101

3

3

138

138

49

3

19

2

5

3

2

2

5

3

1

1

3

60

23

37

17

17

25

8

16

1

21

21

303

95

4

198

6

368

2

359

7

303

33

269

1

3

3

369

5

196

168

55

55

65

65

3

3

10

10

8

8

6

6

20

20

71

7

47

17

1

1

50

50

2

2

4

4

7

3

4

1

1

53

4

49

309

2

154

1

10

1

55

64

3

5

8

6

1

1

107

107

1

1

1

2090

903

8

4

4

166

90

76

726

720

6

3

3

1186

3

3

1112

1112

2

2

69

69

1

1

1

158

158

157

78

72

7

1

1

232

3

3

3

216

216

216

13

13

13

7

5

5

5

2

2

2

800

1

1

1

693

1

1

13

6

7

2

2

380

4

360

16

24

24

1

1

11

1

9

1

50

50

88

88

45

45

13

13

10

10

55

55

13

13

13

1

1

1

62

5

1

4

11

5

3

1

2

26

26

13

3

10

5

5

2

1

1

30

2

2

26

25

1

2

2

12002

968

234

1

1

95

95

2

2

136

3

1

132

734

734

2

3

34

3

15

3

1

2

666

1

4

3275

210

8

8

49

49

31

16

11

4

112

112

2

2

8

3

5

3065

2240

2232

8

14

14

31

31

3

3

285

285

27

27

17

3

14

35

35

381

381

20

5

15

5

5

4

4

3

3

68

49

1

1

48

4

44

19

19

1

1

17

89

89

89

89

53

53

5

1

3

1

46

46

2

2

47

2

2

2

45

45

45

8

8

8

6

2

7494

3

2

2

1

1

2

2

2

1702

1701

15

709

1

3

955

3

7

2

6

1

1

10

10

1

9

5757

299

293

1

5

2150

1074

126

950

60

60

2192

1899

293

9

9

1022

1022

14

14

3

2

1

7

7

1

1

20

18

18

2

2

269

108

108

108

108

161

1

1

1

13

11

11

1

1

1

1

142

24

24

118

111

7

5

5

5

1

1

1

1

1

11

11

11

11

11

8

3

6

6

6

6

6

5

1

96

96

96

96

96

96

5456

7

7

7

7

7

5269

108

32

7

1

3

3

1

1

1

1

2

2

9

1

1

2

3

1

1

12

12

76

73

63

10

3

3

216

214

8

8

5

5

1

1

5

5

4

4

21

21

70

70

31

31

7

7

12

12

7

7

43

43

2

2

2

4742

76

76

76

43

43

43

1663

2

2

3

3

413

413

1245

1245

62

62

62

868

21

21

175

114

61

672

2

8

60

320

7

275

21

1

1

20

1

19

129

48

48

81

81

415

415

10

55

350

11

11

11

339

339

12

18

22

84

180

1

10

1

2

1

8

164

13

13

151

55

13

7

66

1

9

951

82

29

1

52

111

111

111

111

130

41

34

55

517

208

223

23

52

1

10

28

26

12

12

6

6

8

8

2

2

2

172

4

1

1

3

3

56

56

39

13

4

4

1

1

1

1

1

1

1

1

62

62

62

46

4

4

6

2

1

2

1

6

4

2

30

12

4

1

1

1

10

1

3

3

3

3

65

65

65

65

65

1

1

1

1

1

114

114

33

33

7

26

81

14

14

67

67

259

257

11

11

7

7

4

2

2

128

28

28

28

100

12

12

88

88

3

3

3

3

5

5

5

5

6

6

6

6

104

104

104

104

2

2

2

1

1

1

1

1

1

1

1

1

1

195

195

195

195

182

182

13

13

3

3

3

3

3

3

127

127

127

127

127

127

127
